# Find the weakest link. A comparison between demographic, genetic and demo-genetic metapopulation extinction times

**DOI:** 10.1186/1471-2148-11-260

**Published:** 2011-09-19

**Authors:** Alexandre Robert

**Affiliations:** 1 Muséum National d'Histoire Naturelle, Dept. EGB, UMR 7204 CNRS-MNHN-UPMC Conservation des Espèces, Restauration et suivi des Populations, 55 rue Buffon, 75005 Paris, France

## Abstract

**Background:**

While the ultimate causes of most species extinctions are environmental, environmental constraints have various secondary consequences on evolutionary and ecological processes. The roles of demographic, genetic mechanisms and their interactions in limiting the viabilities of species or populations have stirred much debate and remain difficult to evaluate in the absence of demography-genetics conceptual and technical framework. Here, I computed projected times to metapopulation extinction using (1) a model focusing on the effects of species properties, habitat quality, quantity and temporal variability on the time to demographic extinction; (2) a genetic model focusing on the dynamics of the drift and inbreeding loads under the same species and habitat constraints; (3) a demo-genetic model accounting for demographic-genetic processes and feedbacks.

**Results:**

Results indicate that a given population may have a high demographic, but low genetic viability or vice versa; and whether genetic or demographic aspects will be the most limiting to overall viability depends on the constraints faced by the species (e.g., reduction of habitat quantity or quality). As a consequence, depending on metapopulation or species characteristics, incorporating genetic considerations to demographically-based viability assessments may either moderately or severely reduce the persistence time. On the other hand, purely genetically-based estimates of species viability may either underestimate (by neglecting demo-genetic interactions) or overestimate (by neglecting the demographic resilience) true viability.

**Conclusion:**

Unbiased assessments of the viabilities of species may only be obtained by identifying and considering the most limiting processes (i.e., demography or genetics), or, preferentially, by integrating them.

## 1. Background

The role of genetic deterioration in the extinction of endangered species or populations has long been controversial. For over 20 years, several authors have proposed that most populations go extinct for environmental or demographic reasons before genetic deterioration will affect them [1,2]. Yet, theories predict that the properties of most threatened populations (reduced size, isolation, fragmentation) should lead to inbreeding depression [3], mutation accumulation [4] and loss of evolutionary potential [5]. All these expectations have been empirically verified [6-8] and the important role of genetic deterioration processes in population declines and extinctions has been demonstrated by empirical evidence [9,10] and extensive analyses [11]. In spite of these lines of evidences, the weight of genetic deterioration mechanisms in limiting population viability remains difficult to evaluate, mainly because the classical approaches used to assess the "demographic" and "genetic viabilities" are different and neglect the interactions between demographic and genetic processes. In the field of conservation biology, population viability analyses (PVAs) are generally based on demographic analysis, use species or population specific data, and apply emphasis to stochastic processes. Although more and more demographic PVAs include genetic considerations (60% of published PVAs, [12]), most of these models focus on the effect of inbreeding depression only, with no possibility for selection to be accounted for mechanistically, and using inappropriate generic estimates of lethal equivalents.

On the other hand, genetically based viability assessments generally neglect realistic ecological constraints (such as variation in population size or environmental quality). Contrary to demographic ones, insights on the "genetic viability" of species or populations use generic (not specific) data and come from two main categories of studies: (i) genetically determined minimum viable population sizes (MVPs), corresponding to the size necessary to compensate the loss of quantitative genetic variation (through genetic drift) by gains through mutations [13]; (ii) theoretical, metapopulation scale studies focusing on the dynamics of deleterious mutations and their effects on fitness [14].

Since the first formulation of the demographic-genetic (hereafter, demo-genetic) interactions [15], a few theoretical studies have examined the viability of (meta)populations by considering the dynamics of the drift and inbreeding loads as well as explicit and realistic demographic and environmental constraints [16-20] and no study has so far systematically and theoretically compared demographic, genetic and demo-genetic viabilities in realistic (variable) environments. Besides the fundamental importance of understanding how ecological and evolutionary proximal factors limit species viability (e.g., the need of theoretical arguments to solve the debate on the role of genetic deterioration referred above), a general assessment of the conditions under which ecological or genetic factors are the most critical to viability might have considerable applications in the definition of species or population conservation status [21]. Important fields of application are (i) the definition of MVPs, which should differ according to whether ecological [22] or genetic [13] processes are considered. Recent examples include demo-genetic modeling of lake sturgeon populations, indicating that MVP estimates are strongly underestimated if the effects of inbreeding are neglected [23]; (ii) solving potential antagonism between genetic and demographic aspects in metapopulations [24] and considering genetic connectivity in reserve design (see an example on the jaguar in ref. [25]) (iii) help defining optimal strategies in conservative restoration or supplementation programs [26,27]. For example, recent demo-genetic study on *Centaurea corymbosa*, a narrow-endemic plant, provided guidelines for future reintroductions of the species regarding the number of seeds required and their initial distribution in space to limit the demographic effects of genetic self-incompatibility [28].

Here, I compute projected times to metapopulation extinction using three different stochastic simulation models: (i) a demographic model assuming that population growth is finite and has a constant expectation; (ii) a genetic model accounting for variation in the drift and inbreeding loads. In this model, patch population sizes are assumed always equal to local carrying capacities. However, carrying capacities may vary through time in response to environmental variations, with subsequent effects on the dynamics of genetic loads. The metapopulation is assumed genetically extinct if the average genetic load reaches a certain threshold (i.e., when the overall population becomes deterministically decreasing); (iii) a demo-genetic model accounting for demographic (finite growth rate) and genetic processes (accumulation of loads) as well as demo-genetic feedbacks. Similar environmental and intrinsic constraints were applied to these systems and the demographic, genetic and demo-genetic extinction times that they provided were compared.

## 2. Results

### General relationships between ecological metapopulation settings and extinction times

Comparisons between exponential and Weibull survival models suggested that all extinction rates increased with time (see Additional file 1), so only Weibull models were considered in subsequent survival analysis. Comparisons among regression models indicated that (i) the models including all scenarios (survival models) and those including only the scenarios for which all trajectories were extinct after 1,000,000 generations (hereafter referred to as the subset of *extinct trajectories*) are in excellent agreement; (ii) the effects of ecological variables on demographic (*T_D_*) and genetic (*T_G_*) viabilities are qualitatively similar. In all cases, *T_D _*and *T_G _*were positively related to dispersal rate, fecundity and total carrying capacity, while they decreased with fragmentation, as well as the frequency of perturbations (but see below for more details on the effects of fragmentation). The spatial correlation of environmental perturbations (i.e., *C_p _*= 1) always reduced demographic viability (as compared with the case where *C_p _*= 0) but had weaker and more complex effects on genetic viability.

Although ecological variables had similar qualitative effects on the viabilities *T_D _*and *T_G_*, the strengths of these effects differed considerably between the demographic and genetic models. A hierarchical partitioning (HP) indicated that *T_G _*was mainly related to metapopulation size and fragmentation (*K_t_*, R^2 ^= 51%; *N*, R^2 ^= 18%) while *T_D _*mostly depended on the basic growth rate and the perturbation regime (*F*, R^2 ^= 28%; *P*, R^2 ^= 17%) (All detailed results are provided in Additional file 1).

**Figure 1:**
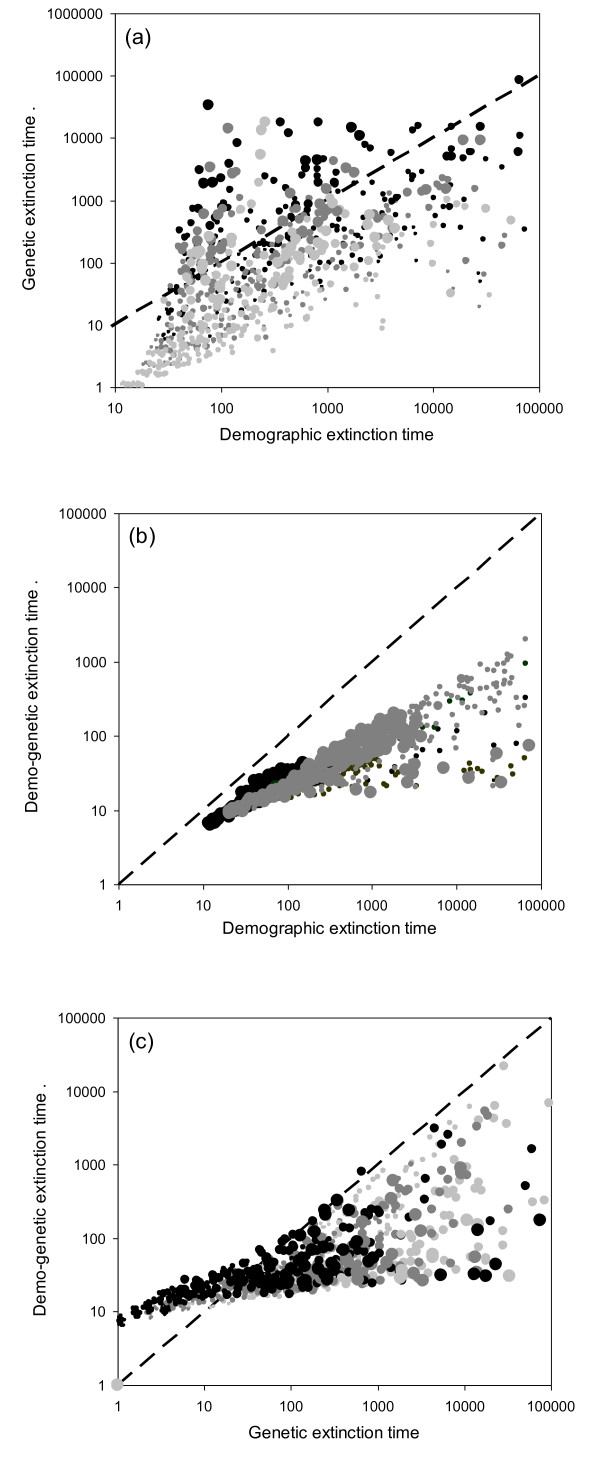
**Comparisons among demographic, genetic and demo-genetic extinction times**. In all three panels, each circle represents the median extinction time computed from 250 trajectories for a given ecological scenario. Dotted lines are the bissectrices. (1a) Demographic versus genetic median extinction times. The size of symbols is proportional to overall metapopulation size (*K*_t_); lighter colors indicates higher levels of fragmentation (black: *N *< 5; dark grey: 5 ≤ *N *≤ 10; light grey: *N *> 10). (1b) Demographic versus demo-genetic median extinction times. The size of symbols is related to the regime of perturbations (large (small) symbols = high (low) frequency of perturbations). The color of symbols indicates the basic fecundity rate (grey: *F *= 1.5; black: *F *= 1.1). (1c) Genetic versus demo-genetic median extinction times. The size of symbols is proportional to overall metapopulation size (*K*_t_); lighter colors indicates higher levels of fragmentation (black: *N *< 5; dark grey: 5 ≤ *N *≤ 10; light grey: *N *> 10).

Therefore, when considering all *extinct trajectories *scenarios, demographic and genetic extinction times were only moderately correlated (Kendall's τ = 57%), but correlation increased when considering only scenarios with a constant environment (τ = 78%). In most situations, the demographic extinction time (T_D_) was longer than the genetic extinction time T_G _(median of T_G_/T_D _= 0.17) but the distribution of the T_G_/T_D _ratio was wide and skewed to the right (Mean = 2.1, Min = 7.10^-5^, Max = 430). Further analysis indicated that this ratio strongly increases with total population size and the frequency of perturbations level and decreases with fragmentation (HP: R^2 ^were equal to 44%, 23% and 6% respectively for *K_t_, N *and *P*, see Additional file 1). Hence, the ecological scenarios for which the genetic viability was higher than the demographic viability were those assuming large metapopulations and low levels of fragmentation (main effects are summarized on Figure 1a).

### Comparisons between demographic and demo-genetic extinction times

As expected, adding genetic constraints to the demographic model always decreased viability. However, the ratio (T_DG_/T_D_) varied strongly, from 10^-4 ^to 0.6 (Median = 0.15, Mean = 0.19, SD = 0.15). Multiple regressions and HP indicated that this ratio was mostly influenced by the perturbation regime and the basic fecundity (*P*, R^2 ^= 23%; *F*, R^2 ^= 7%, details in Additional file 1). The demographic and demo-genetic extinction times were very close to each other in slow growing metapopulations with a highly variable environment. In other situations, T_DG _may be several orders of magnitude lower than T_D _(Figure 1b).

A univariate regression model indicated that T_D _explains about 68% of the variance in T_DG_. The residuals of this regression were positively correlated with the metapopulation carrying capacity (HP, R^2 ^= 43%, Additional file 1).

### Comparisons between genetic and demo-genetic extinction times

Surprisingly, adding demographic constraints to the genetic model reduced viability in only 66% of the scenarios investigated (the ratio T_DG_/T_G _ranged from 7.10^-5 ^to 8.3 with a median value of 0.47). The analysis indicated that this ratio was mostly influenced by population size and fragmentation (*K_t_*, R^2 ^= 9.1%, *N*, R^2 ^= 5.8%, Additional file 1). In small and highly fragmented metapopulations, the genetic extinction time was shorter than the demo-genetic extinction time while the reverse was generally true in other cases (Figure 1c, Additional file 1).

### *Use of *T_D _*and *T_G _*to estimate *T_D*G*_

When considered as single predictors in *extinct trajectories *scenarios, T_D _and T_G _explained respectively 68% and 65% of the variance in the demo-genetic time to extinction. Using the maximum value (between T_G _and T_D_) did not improve model fit (R^2 ^= 64.6%), while using the minimum value increased the R^2 ^to 78%, a value close to the R^2 ^obtained by including both T_G _an T_D _in the model (without interaction). The interaction term between T_G _and T_D _was positive and highly significant but only marginally improved model fit (R^2 ^= 80%).

### Complementary analyses

A complementary analysis indicated that the type of environmental perturbations considered does not influence the qualitative effects of ecological variables on T_D _and T_G _and T_DG_, although the quantitative effects of *F *and *P *on T_D _may vary with the type of perturbations (Additional file 1).

### A focus on the effect of fragmentation on extinction times

**Figure 2:**
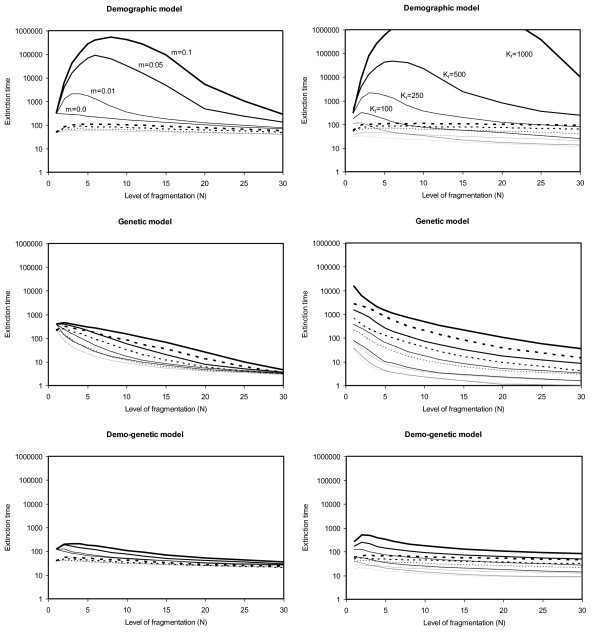
**Demographic, genetic and demo-genetic median extinction times as functions of the level of metapopulation fragmentation (*N*)**. Extinction times are presented for different dispersal rates (*m*, ranging from 0 to 0.1, *K_t _*fixed to 250, left panel) and different overall metapopulation carrying capacities (*K_t_*, ranging from 50 to 1000, *m *fixed to 0.01, right panel). Continuous lines: low frequency of environmental perturbations (*P *= 0.05); dotted lines: high frequency of environmental perturbations (*P *= 0.15). In all cases, environmental perturbations occur and act independently among patches. *F *= 1.1.

The effect of the level of fragmentation (*N*) on demographic, genetic and demo-genetic viabilities is illustrated in Figure 2. The comparison between demographic and genetic viabilities revealed contrasting patterns. With the demographic model, the optimal level of fragmentation increased with overall population size and dispersal rates and could reach high levels (> 10-15 patches). In contrast, genetic viability was maximized with low fragmentation levels (1 or 2 patches) in all situations. In this context, the consideration of demo-genetic interactions tended to buffer extinction times and all demo-genetic optimal fragmentation levels were intermediary between optimal demographic and genetic levels. These patterns remained qualitatively as long as environmental perturbations are spatially independent (sensitivity analyses presented in Additional files 2, 3 and 4).

## 3. Discussion

### Similarities and differences in demographic and genetic extinction patterns

Most ecological settings considered here had similar qualitative effects on demographic and genetic viabilities, in agreement with theoretical expectations. Large population sizes allow limiting demographic stochasticity [29] as well as the inbreeding and drift loads [30]; high dispersal rates are associated with demographic rescue and recolonization [31] and reduced drift load [30]; perturbations cause population bottlenecks that are directly associated with demographic extinction and inflate the genetic load [32]; high fecundity rates allow counteracting the effect of demographic and environmental stochasticities [33] and elevate the genetic extinction load threshold. Only fragmentation (*N*) has less obvious effects (see below). However, despite these similarities, the processes leading to "demographic" and "genetic" extinctions are fundamentally different. Demographic viability is related to the mean and variance of the rate of increase [29], which are primarily influenced by the perturbation regime and the basic growth rate (*F *and *P*). In contrast, genetic viability is associated with the inbreeding and drift loads, themselves primarily related to population size and fragmentation (*K_t _*and *N*, [14,30]). The causes and implications of this are further developed below.

### Demographic processes and hard selection models

While the genetic model presented here is a soft selection model (i.e., each local population contributes to the next generation independently of its mean relative fitness), the integration of demographic processes (i.e., the demo-genetic model) clearly adds a hard selection component (since the size and contribution of demes partly depend on their genetic loads). Theoretical work predicts that (1) With soft selection, population structure should reduce the efficiency of selection against mildly deleterious, nearly additive mutations [34] but will favor selection against severe and highly recessive mutations [35]; (2) With hard selection, structure should lead to reduced loads even with additive or nearly additive mutations [14].

While the present results are in agreement with predictions from soft selection models, the beneficial genetic effects of population structure expected under hard selection are not visible with the present demo-genetic model (i.e., increasing *N *and/or decreasing *m *has a negative effect on viability). This discrepancy is due to the fact that (i) the demo-genetic model is only a partial hard selection model, as local population size primarily depends on regulation processes (independent from genetic loads); (ii) potential genetic effects are masked by demographic effects (see, in particular, Figure 2c); (iii) the genetic and demo-genetic viabilities expressed in the present paper do not necessarily reflect expected equilibrium patterns. In particular, the purging processes leading to reduced inbreeding or drift load may be demographically costly, which implies that some metapopulations will have a low demo-genetic viability despite a small expected equilibrium load.

### Why genetic models are insufficient to estimate viability

It is generally admitted in the theoretical literature that ecology-genetic interactions should strongly influence the persistence time of populations [15-18,36,37]. The demo-genetic interaction refers to the fact that the occurrence of genetic processes is affected by the demographic state of the population, and vice versa, leading to synergistic or antagonistic effects on viability. Here, the process of demo-genetic extinction may be decomposed in two phases: an *accumulation *phase, during which the overall genetic load increases while population size remains approximately equal to the carrying capacity, and an *extinction *phase during which the population declines to extinction. Demo-genetic interactions are important in both phases: (1) during the *accumulation *phase, stochastic variations in population size decrease the effective size and accelerate the rise of the genetic load; (2) during the extinction phase, the population decline increases the rate of mutation accumulation, which in turns accelerates population decline (the so-called mutational meltdown, formalized and discussed in refs [15,16]).

The *extinction *phase (i.e., phase 3 in Lynch and colleagues papers) is generally assumed much shorter than the *accumulation *phase in theoretical genetic models, and the extinction time is assumed equal to the duration of the *accumulation *phase (i.e., the time necessary to obtain a deterministically decreasing population). Here, in all scenarios, the initial decrease in fitness was more rapid with the demo-genetic model as compared with the genetic model (see Additional file 5). This led to T_DG _< T_G _in two thirds of the scenarios investigated, in agreement with previous findings on the demo-genetic interaction. However, in the other third of the scenarios, demo-genetic viability was higher than genetic viability (Figure 1c). This comes from the fact that populations may be demographically resilient and persist several generations after their growth rate has become negative (Additional file 5). Therefore, fitness-based estimations of viability may lead to strong over- or under estimations of the risk of extinction, depending of the relative weights of the demo-genetic interaction and the demographic resilience on viability.

### Why demographic models are insufficient to estimate actual viability

As expected, incorporating genetic considerations to the demographic model was unequivocally deleterious. However, the magnitude of the reduction in viability was highly variable. It is generally admitted that the proportional effect of genetic deterioration on viability should be larger for populations with a high demographic viability [17,37-39]. While the present results are in clear agreement with this expectation, they highlight the critical need to distinguish the intrinsic and extrinsic ecological threats to population viability to estimate the effect of genetic problems on extinction. In some of the scenarios investigated, the demo-genetic extinction times were about half the demographic extinction times, while in other cases they were 10,000 times shorter. Typically, the first situation corresponds to large populations with highly variable, low quality environments (environmental stochasticity is the primary cause of extinction, with or without genetic deterioration), while the later corresponds to small, fragmented populations in stable, good quality environments (genetic deterioration is the primary cause of decline and extinction). In the context of the debate over the environmental *versus *genetic causes of species extinction, the present results demonstrate that the arguments of the partisans of the environment [1,2,40] and genetic [9-11] hypotheses are both theoretically justified. The net impact of genetic deterioration processes on extinction may be strongly variable among and within species, since it strongly depends on the ecological conditions faced by metapopulations (although it is clear that some variation in other factors such as mating systems, life history traits, dispersal pattern or demographic history, will further increase this variability). Importantly, however, many human induced environmental changes are likely to engender situations where genetic deterioration is the primary extinction cause, in particular where available habitats have been reduced in quantity, and not in quality.

### The case of fragmentation

Contrary to other ecological settings (*K_t_, m*,...), fragmentation has non obvious (and potentially contradictory) qualitative effects on demographic and genetic viabilities. From a genetic view-point, most theoretical studies agree that subdivision has detrimental effects on fitness (i.e., few large patches perform better than many small patches [17,19,30]). In the ecological literature, conclusions are less clear [41]. The optimal level of fragmentation depends on overall population size and dispersal ([42-44], see upper panels of Figure 2) as well as on the regime of perturbations [45,46]. In the absence of fragmentation (single population), strong perturbations may rapidly drive the whole population to extinction, even if population size is large [33,47]. In contrast, with very high levels of fragmentation (many small populations), environmentally induced extinction risk is spread over several units, but the probability of local extinction due to demographic stochasticity increases as local population sizes decrease. Thus, in many realistic situations, an intermediate level of fragmentations will be optimal. Theoretical work indicates that this general result remains true under a wide range of realistic conditions, provided that (i) the cost of dispersal is not too strong; (ii) environmental variations are not fully correlated among patches of habitats [43,45,46].

Consequently, (i) demographically optimal levels of fragmentation may be much higher than genetically optimal levels; (ii) demo-genetic optimal levels are, in most cases, intermediary between demographic and genetic optimal levels (Figure 2). This implies that optimal levels of fragmentations (e.g., in the context of reserve design) may be either under- or over-estimated when based on simple demographic or genetic approaches.

## 4. Conclusion

Understanding the fine mechanisms of extinction is essential to the study of evolution and has crucial implications in the context of the current human induced mass extinction crisis [48]. In the field of conservation biology, the definition of species or population conservation status is generally achieved by considering both ecological and genetic constraints [21]. However, there is no framework to hierarchise these constraints. For example, when assessing extinction risk of a given population, should one give priority to the demographically based or genetically based estimate? The more pessimistic one? Something intermediary between both? These questions are even more important when considering situations where demographic and genetic assessments lead to contradictory management strategies, e.g., in reserve design [24] or supplementation programs [26].

The present results indicate that (i) in most cases, the demo-genetic extinction time is lower than both the demographic and genetic extinction times, due to a demo-genetic interaction; (ii) while the demo-genetic extinction time is weakly correlated with demographic and genetic extinction times, it is substantially more correlated with the minimum between them, suggesting that considering the most limiting factors (demographic or genetic) to estimate population viability is a reasonable approach; (iii) a given population may have a high demographic, but low genetic viability or vice versa.

At the evolutionary and ecological scales, most species extinctions are ultimately caused by changes in their environment. However, particular environmental constraints may primarily affect either demographic, genetic processes, or both of them. An illustration of this point is the contrast between captive and wild threatened populations. Small captive populations face important deleterious evolutionary changes (inbreeding depression, loss of evolutionary potential, adaptation to captivity...) but are demographically safe (benign and buffered environment). On the other hand, wild populations with larger carrying capacities and sizes suffer less genetic problems but are ecologically vulnerable (reduction of environmental quality, increase of environmental variance). While this antagonism is addressed in recent work in the particular context of the wild/captive population systems [49], there is still no general framework to evaluate how various constraints affect demographic and genetic possesses, and to estimate their respective weights in limiting the persistence of species. The present results indicate that, although most environmental constraints have similar qualitative effects on genetic and demographic viabilities, direct threats or constraints associated with reductions in habitat quality (e.g., exploitation, pollution, increase of climatic variance,...) will primarily decrease demographic viability, whereas reduction in habitat quantity (e.g., fragmentation or loss of habitat following change in land-use or climate,...) will primarily affect genetic processes.

## 5. Methods

I used a monoecious, individual-based model with discrete generations to describe the dynamics of a metapopulation with *N *patches and a total carrying capacity *K_t_*.

### Selected genetic variation

The genome of each individual was explicitly represented as *L *= 1000 different diploid loci that could carry either a wild-type or a deleterious allele. Following the approach of Jaquiéry *et al*. [19], I considered variable selection (*s*) and dominance (*h*) coefficients among loci and a negative correlation between them [50]. Selective coefficients were exponentially distributed among the *L *loci, with an average severity set to *s *= 0.05 [51]. At each locus *k*, the dominance coefficient *h_k _*was computed as *h_k _*= exp(-*cs_k_*)/2, where *c *was a constant computed so that the expected dominance of all mutations in the genome equaled *h *= 0.35 [19,52]. During fertilization, the probability of transmission of each allele at each locus was given by Mendelian rules. New deleterious mutations stochastically occurred in each zygote (Poisson distributed, with mean *U *= 1.0, [53]). I assumed no epistasis, no linkage and no reverse mutations. Importantly, the mutation parameters used in the present study derive from model organisms, such as *Drosophila*, nematodes or bacteria. These estimates are used in most theoretical studies, and should be viewed as conservative estimates (in particular, per zygote mutation rates are likely to be higher in most species of conservation concern than in *Drosophila *species, [51,54]).

At each locus *k*, the initial frequency of deleterious alleles was given by the mutation-selection balance [55]: *q*_0*k *_= *U*/(*L s_k _h_k_*). The genome of the initial population considered at *t *= 0 was implemented according to these frequencies (Bernoulli process). Thus, the individuals present at *t *= 0 derived from a large panmictic population (for all ecological scenarios investigated, I assumed that sudden reduction and fragmentation of the habitat occurred at *t *= 0).

I assumed that deleterious alleles acted on offspring viability. The proportional reduction in survival of the individual *i *was then given by

$$w_{i}=\frac{1}{w_{0}}\overset{L}{\underset{}{\prod }}w_{ki}$$

Where *w_ki _*was equal to 1, 1-*h_k_s_k _*or 1-*s_k_*, for a locus *k *without mutation, with a heterogeneous deleterious mutation, or a homozygous mutation, respectively. *w*_0 _was the expected initial reduction in fitness due to deleterious alleles present at time zero, given by

$$w_{0}=\overset{L}{\underset{}{\prod }}{\left(1-h_{k}s_{k}\right)}^{q_{0k}}$$

In each generation *t*, the average reduction in survival of the metapopulation (*W*_(t)_) was computed.

### Demo-genetic model

In each generation *t*, in each patch *j*, all individuals paired randomly with possibility of self-fertilization at the random rate. The fecundity of each individual was determined by a Poisson trial of parameter *F*. Punctual negative environmental perturbations resulted in a reduction (punctual in time) of local offspring survival. The survival of each offspring *i *present in patch *j *at generation *t *was drawn from a Bernoulli function of expectation *s*_0*i *_= (1-*p_*j*(*t*)_*)*w_i_*, that depended on the genetic characteristics *w_i _*of the individual (see above) and on the occurrence of a local perturbation. The occurrence of a perturbation was determined by a Bernoulli trial of parameter *P *(= the general per generation frequency of perturbations). If no perturbation occurred in patch *j *at time *t, p*_*j*(*t*) _was set to zero. If a perturbation occurred, the value of *p*_*j*(*t*) _was drawn from an empirical severity distribution [47]. The parameter *C_p _*denoted the degree of spatial correlation of perturbations. I considered two extreme situations where perturbations were either independent (*C_p _*= 0) or fully correlated in time (*C_p _*= 1) across patches. An alternative method to model perturbations (in which perturbations temporally reduce local carrying capacity) is presented in results and Additional file 1.

I assumed that all patches had the same maximum local carrying capacity (*K = K_t_/N*), and were initially at their carrying capacity. All parents died after reproduction. Local regulation consisted in a truncation of population size of the offspring to *K *in each generation for each patch. Truncation was made independently of the genetic qualities of individuals.

Dispersal occurred by assuming that a proportion *m *(Bernoulli process) of the individuals present in each patch after the selection and regulation steps (but before the reproduction step) emigrated (island model) in each generation. Extinction occurred when there were no more individuals in the metapopulation.

### Genetic model

In each generation *t*, in each patch *j*, two parents were randomly selected to reproduce. Fertilization and new mutations occurred as described above to create the new individual *i*, that survived or not, according to its relative fitness *w_i _*(Bernoulli process). This operation (random selection of parents with replacement) was repeated until there was *K*_*j*(*t*) _surviving offspring. Then all parents died. *K_j_*_(*t*)_, the carrying capacity of each patch *j *at generation *t*, depended on the occurrence of a perturbation in patch *j*. It was computed as the rounded value of *K*(1-*p*_*j*(*t*)_), where *p*_j(*t*) _was as described above for the demo-genetic model. In case where all local carrying capacities equaled zero, one patch was randomly drawn and its carrying capacity was set to one, so that the overall carrying capacity was always higher than zero. Here, contrary to the demo-genetic model, (i) population dynamics were independent from *W*_(*t*) _and *F*. Local populations were always at their carrying capacity, which could vary for environmental reasons as in the demo-genetic model: perturbations were modeled by the parameters *P *(= frequency of perturbations) and *C_p _*(= spatial correlation of perturbations) and acted by temporally reducing local carrying capacities (same distribution of severity as in the demo-genetic model); (ii) "demographic extinction" could not occur (there was always at least one individual in the metapopulation). Environmentally-driven variations of population size (i.e., environmental perturbations) tended to reduce the long term effective size and increase the inbreeding level, with subsequent effects on selection. The metapopulation was assumed "genetically extinct" when the average offspring survival *W*_(*t*) _dropped below the threshold W* = *F*^-1^, where *F *was the average basic fecundity (corresponding to the theoretical population's replacement rate in the absence of genetic load). Dispersal occurred as for the demo-genetic model.

### Demographic model

The demographic model worked similarly to the demo-genetic model, but the effect of selected genetic variation on population dynamics was removed (i.e., *w_i _*was set to one for all individuals). The similarities and differences between the demographic, genetic and demo-genetic models are summarized in Table 1.

**Table 1:** Modalities of population processes for the demographic, genetic and demo-genetic models

Process	Demographic model	Genetic model	Demo-genetic model
Reproduction	Fecundity *F *for all individuals	Fecundity *F *for all individuals	Fecundity *F *for all individuals
Survival of offspring	*w_i _*= 1 for all individuals	*w_i _*depends on genetic characteristic of *i*	*w_i _*depends on genetic characteristic of *i*
Local intrinsic dynamics	Results from stochastic realizations of fecundity and offspring survival	Local populations always at their carrying capacity	Results from stochastic realizations of fecundity and offspring survival
Selection	No variance in fitness	Based on *w_i_*	Based on *w_i_*
Regulation	Local truncation to *K*	Local populations always at their carrying capacity *K*	Local truncation to *K*
Dispersal	Emigration rate *m *(Bernoulli process)	Emigration rate *m *(Bernoulli process)	Emigration rate *m *(Bernoulli process)
Modalities of perturbations	Reduce either offspring survival of local carrying capacity	Reduce local carrying capacity	Reduce either offspring survival of local carrying capacity
Severity of perturbations	Empirical distribution [47]	Empirical distribution [47]	Empirical distribution [47]
Spatial correlation of perturbations	Either fully correlated or uncorrelated	Either fully correlated or uncorrelated	Either fully correlated or uncorrelated
Extinction	When *Size *= 0	When *W *= *F*^-1^	When *Size *= 0

*F *: fecundity per individual; *w*_i _: juvenile survival for individual *i *; *W *: average offspring survival; *m *: emigration rate; *K *: local carrying capacity; *Size *: total metapopulation size.

### Simulation protocol

Demographic, genetic and demo-genetic metapopulation viabilities were examined by assuming that time zero corresponded to sudden environmental changes (reduction and fragmentation of the habitat). I used Monte Carlo simulations for 1476 combinations of the ecological input parameters (*N, K_t_, m, F, P, C_p_*, see Table 2 for details). For each combination, 250 population trajectories were drawn. In each case, the median times to demographic, genetic and demo-genetic extinction were computed (respectively noted *T_D_, T_G _*and *T_DG_*). I was interested in investigating the viability of small to medium metapopulations (*K_t _*ranged from 50 to 2000) with a wide range of persistence times. With the demo-genetic model (i.e., the most realistic one), typical persistence times were a few tens or hundreds of generations (median value was less than 100 generations); however, in some scenarios assuming large populations with high and stable growth, times to extinction could be very long, especially with the demographic or genetic sub-models. Therefore, for technical reasons, all trajectories were stopped after 1,000,000 generations. In scenarios for which one or several trajectories were non extinct after 1,000,000 generations, extinction times could not be computed. These extinction times were treated either as right-censored data (i.e., data for which extinction was not observed, [56]) or as lacking data in statistical analyses (see details below).

**Table 2:** Values of ecological input parameters used to generate the 1476 combinations used in statistical analyses

Parameter	Values
*N*	1, 3, 5, 10, 20, 30
*K_t_*	50, 100, 250, 500, 1000, 2000
*m*	0, 0.01, 0.05, 0.1
*F*	1.1, 1.5
*P*	0, 0.05, 0.15
*C_p_*	0, 1

*N*: number of patches; *K_t_*: total carrying capacity of the metapopulation; *m*: dispersal rate; *F*: individual fecundity; *P*: per generation frequency of environmental perturbations; *C_p_*: spatial correlation of perturbations.

### Statistical and graphical analysis

I used survival models, generalized linear models (GLM) and hierarchical partitioning (HP) to examine the relationships between ecological input parameters and extinction times.

As a first step, exponential and Weibull survival models assuming right censored extinction events were fitted to examine the relationship between extinction times (*T_D_, T_G _*and *T_DG_*) and ecological input parameters, considering all 1476 scenarios. The Weibull and exponential models were compared to describe whether extinction rates were constant over time or not [57].

As a second step, GLM and HP were applied to those scenarios for which extinction times were available (i.e., excluding scenarios for which one or several trajectories were non extinct after 1,000,000 generations) to examine variations in the ratios of extinction times (e.g., *T_D_*/*T_G_*) and their relationships with ecological variables. HP uses all models in a regression hierarchy to distinguish those variables that have high independent correlations with the dependent variable. At all steps, results from the survival models, GLM and HP were compared to help interpretation. Although most extinction events occurred within the range 10-10,000 generations (> 80% of scenarios), the variance was high and the distribution of extinction times was non-normal. Thus, graphical results are presented using logarithmic scales and extinction times were log-transformed (Neperian logarithm) in all statistical analysis. Other dependent and independent variables were transformed using usual functions to achieve the best linearity (transformations provided in results). The aim of these analyses was not to test the significativity of regressions but rather to examine the direction and strength of relationships between ecological variables and viabilities [20]. All qualitative results were compared to theoretical expectations and in cases where results were different from expectations, additional simulations were run to determine the underlying causes of observed patterns. Metapopulation models were developed in Pascal language (source codes are available upon request). All statistical analysis was performed with R 2.10.1 [58], specifically using the Survival [57] and Hier.part [59] packages.

## Supplementary Material

Additional file 1 **Linear relationships between ecological parameters and viability metrics**. Contains details on statistical analysis of model outputs. Click here for file

Additional file 2 **Sensitivity of fragmentation results to genetic parameters (U, s and h)**. Click here for file

Additional file 3 **Effect of fragmentation on extinction times: regime of spatially correlated perturbation**. Click here for file

Additional file 4 **Effect of fragmentation on extinction times: complementary results (use of an alternative protocol to model environmental perturbations)**. Click here for file

Additional file 5 **Fitness and population size reductions: an illustration**. Click here for file
