# Supplementary material for: Find the weakest link. A comparison between demographic, genetic and demo-genetic metapopulation extinction times
Source: BMC Evol Biol. 2011 Sep 19;11:260. doi: 10.1186/1471-2148-11-260 (PMC3185286; doi:10.1186/1471-2148-11-260)
Supplement: Additional file 1 — Linear relationships between ecological parameters and viability metrics. Contains details on statistical analysis of model outputs. [file 1471-2148-11-260-S1.PDF]

Additional file 1: Linear relationships between ecological parameters and viability metrics

I. Linear relationships between times to extinction (T_D, T_G and T_{DG}) and ecological input data

In the following Survival model tables, estimates of the coefficients of regression are provided with standard errors (*sd*), Z statistics (Z) and p-value (*P*). In generalized linear model (GLM) tables, estimates of the coefficients of regression are provided with standard errors (*sd*), Student statistics (*T*) and p-value (*P*). In hierarchical partitioning (HP) tables, the independent proportion of variance explained by each explanatory variable (R^2) is provided with a p-value (P) obtained from a randomization test (1000 iterations, see Mac Nally 2002, Walsh & Mac Nally 2003).

The dependent variables were the demographic (T_D), genetic (T_G) or demo-genetic (T_{DG}) median times to extinction. Explanatory variables were the dispersal rate (*m*), the overall carrying capacity (K_t), number of patches (*N*), individual fecundity (*F*), per generation frequency of perturbations (*P*) and coefficient of correlation of perturbations among patches (C_p). Interactions between explanatory variables were not considered. In all cases, T_D, T_G and T_{DG} were log-transformed. Other data transformations are directly indicated in tables.

1) Constancy of extinction rates over time

Exponential and Weibull survival models assuming right censored extinction events were fitted to examine the relationship between extinction times and ecological input parameters, considering all 1476 scenarios (Cox and Oakes 1984). The Weibull and exponential models were compared to describe whether extinction rates were constant over time or not (Crawley, 2007). The analysis indicated that extinction rates were not constant across time for the demographic (T_D), genetic (T_G) and demo-genetic (T_{DG}) median times to extinction (Table I.1.1 below). In all cases, extinction rates increased with time (the log(scale) estimate was significantly different from zero and negative in all Weibull regressions, see Tables I.2.1, I.3.1 and I.4.1 below). All subsequent survival models (subsections 2 to 4) are based on a Weibull distribution of extinction times.

Table I.1.1 Comparison between exponential and Weibull models (ANOVA) for T_D, T_G and T_{DG}.

Dependant variable	χ^2	d.f.	P
T_D	847.1394	*1*	$<10^{-4}$
T_G	253.7302	*1*	$<10^{-4}$
T_{DG}	767.465	*1*	$<10^{-4}$

2) The demographic time to extinction (T_D)

*Table I.2.1 Relationships between ecological input data and Log(**T_D**) (Survival model)*

n=1476 datasets; model log-likelihood=-2068.5; χ^2=1691.18; d.f.=6; P<10^{-4}

Variable	Estimate	*sd*	*Z*	*P*
m	2.67	0.34	7.83	$<10^{-4}$

N	-0.01	0.00	-4.19	$<10^{-4}$
Log(K_t)	0.17	0.01	16.06	$<10^{-4}$
F	1.39	0.07	20.68	$<10^{-4}$
P	-9.68	0.22	-44.87	$<10^{-4}$
C_p	-0.41	0.03	-15.47	$<10^{-4}$
Log(scale)	-1.07	0.03	-37.51	$<10^{-4}$

*Table I.2.2 Relationships between ecological input data and Log(**T_D**) (GLM)*

n=770 datasets, overall adjusted R^2=0.69

Variable	Estimate	*sd*	*T*	*P*
m	9.01	1.13	8.00	$<10^{-4}$
N	-0.05	0.00	-12.81	$<10^{-4}$
Log(K_t)	0.82	0.03	23.69	$<10^{-4}$
F	6.07	0.22	27.66	$<10^{-4}$
P	-18.26	0.87	-21.07	$<10^{-4}$
C_p	-0.75	0.09	-8.38	$<10^{-4}$

*Table I.2.3 Relationships between ecological input data and Log(**T_D**) (HP)*

n=770 datasets

Variable	R^2	*P*
m	0.002	0.01
N	0.15	$<10^{-4}$
Log(K_t)	0.07	$<10^{-4}$
F	0.17	$<10^{-4}$

P	0.28	$<10^{-4}$
C_p	0.004	0.001

3) The genetic time to extinction (T_G)

Table I.3.1 Relationships between ecological input data and Log($\boldsymbol{T_G}$) (Survival model)

n=1476 datasets; model log-likelihood=-2597; χ^2=1352.8; d.f.=6; $P<10^{-4}$

Variable	Estimate	*sd*	*Z*	*P*
m	6.03	0.56	10.80	$<10^{-4}$
N	-0.03	0.00	-14.40	$<10^{-4}$
Log(K_t)	0.53	0.02	29.40	$<10^{-4}$
F	1.24	0.11	11.70	$<10^{-4}$
P	-7.48	0.34	-21.90	$<10^{-4}$
C_p	0.73	0.05	15.30	$<10^{-4}$
Log(scale)	-0.44	0.02	-17.80	$<10^{-4}$

Table I.3.2 Relationships between ecological input data and Log($\boldsymbol{T_G}$) (GLM)

n=990 datasets, overall adjusted R^2=0.87

Variable	Estimate	*sd*	*T*	*P*
m	17.13	0.77	22.39	$<10^{-4}$
N	-0.11	0.00	-39.05	$<10^{-4}$
Log(K_t)	1.75	0.03	68.37	$<10^{-4}$
F	4.75	0.14	32.75	$<10^{-4}$

P	-4.54	0.50	-9.12	$<10^{-4}$
C_p	-0.39	0.06	-6.39	$<10^{-4}$

*Table I.3.3 Relationships between ecological input data and Log(**T_G**) (HP)*

n=990 datasets

Variable	R^2	*P*
m	0.03	$<10^{-4}$
N	0.51	$<10^{-4}$
Log(K_t)	0.18	$<10^{-4}$
F	0.01	$<10^{-4}$
P	0.12	$<10^{-4}$
C_p	0.01	2.10^{-4}

4) The demo-genetic time to extinction (T_{DG})

*Table I.4.1 Relationships between ecological input data and Log(**T_{DG}**) (Survival model)*

n=1476 datasets; model log-likelihood=-2218.9; χ^2=1751.7; d.f.=6; P$<10^{-4}$

Variable	Estimate	*sd*	*Z*	*P*
m	1.45	0.34	4.32	$<10^{-4}$
N	-0.01	0.001	-4.21	$<10^{-4}$
Log(K_t)	0.14	0.01	13.43	$<10^{-4}$
F	0.80	0.07	11.82	$<10^{-4}$
P	-11.00	0.21	-52.34	$<10^{-4}$
C_p	-0.14	0.03	-5.29	$<10^{-4}$

Log(scale)	-0.92	0.03	-34.10	$<10^{-4}$

Table I.4.2 Relationships between ecological input data and Log($\mathbf{T_{DG}}$) (GLM)

n=927 datasets, overall adjusted R^2=0.74

Variable	Estimate	*sd*	*T*	*P*
m	4.54	0.59	7.66	$<10^{-4}$
N	-0.03	0.00	-11.78	$<10^{-4}$
Log(K_t)	0.63	0.02	34.37	$<10^{-4}$
F	4.01	0.12	34.46	$<10^{-4}$
P	-6.12	0.46	-13.17	$<10^{-4}$
C_p	-0.39	0.05	-8.47	$<10^{-4}$

Table I.4.3 Relationships between ecological input data and Log($\mathbf{T_{DG}}$) (HP)

n=927 datasets

Variable	R^2	*P*
m	NA	ns
N	0.29	$<10^{-4}$
Log(K_t)	0.05	$<10^{-4}$
F	0.06	$<10^{-4}$
P	0.33	$<10^{-4}$
C_p	0.005	0.05

II. Linear relationships between the ratios of the times to extinction and ecological input data

In the following Generalized Linear Model (GLM) tables, estimates of the coefficients of regression are provided with standard errors (*sd*), Student statistics (*T*) and p-value (*P*). In hierarchical partitioning (HP) tables, the independent proportion of variance explained by each explanatory variable (R^2) is provided with a p-value (P) obtained from a randomization test (1000 iterations, see Mac Nally 2002, Walsh & Mac Nally 2003).

The dependent variables were the ratios of the times to extinction demographic (T_G/T_D, T_{DG}/T_D and T_{DG}/T_G). Explanatory variables were the dispersal rate (*m*), the overall carrying capacity (K_t), number of patches (*N*), individual fecundity (*F*), per generation frequency of perturbations (*P*) and coefficient of correlation of perturbations among patches (C_p). Interactions between explanatory variables were not considered. In all cases, T_D, T_G and T_{DG} were log-transformed (i.e., the dependent variables are the ratios of $Log_{(time\ to\ extinction)}$). Other data transformations are directly indicated in tables.

1) The ratio (T_G/T_D)

Table II.1.1 Relationships between ecological input data and Log($\mathbf{T_G}$)/Log($\mathbf{T_D}$) (GLM)

n=695 datasets, overall adjusted R^2=0.78

Variable	Estimate	*sd*	*T*	*P*
m	1.53	0.17	9.09	$<10^{-4}$
N	-0.02	0.00	-27.52	$<10^{-4}$
Log(K_t)	0.21	0.01	38.68	$<10^{-4}$
F	0.11	0.03	3.40	7.10^{-4}

P	1.60	0.12	12.97	$<10^{-4}$
C_p	0.07	0.01	5.78	$<10^{-4}$

*Table II.1.2 Relationships between ecological input data and Log(**T_G**)/Log(**T_D**) (HP)*

n=695 datasets

Variable	R^2	*P*
m	0.02	$<10^{-4}$
N	0.23	$<10^{-4}$
Log(K_t)	0.44	$<10^{-4}$
F	NA	ns
P	0.06	$<10^{-4}$
C_p	0.02	$<10^{-4}$

2) The ratio (T_{DG}/T_D)

*Table II.2.1 Relationships between ecological input data and Log(**T_{DG}**)/Log(**T_D**) (GLM)*

n=770 datasets, overall adjusted R^2=0.41

Variable	Estimate	*sd*	*T*	*P*
m	-0.610	0.072	-8.426	$<10^{-4}$
N	0.001	0.000	4.293	$<10^{-4}$
Log(K_t)	-0.006	0.002	-2.479	0.013
F	-0.166	0.014	-11.769	$<10^{-4}$
P	0.997	0.056	17.919	$<10^{-4}$
C_p	0.051	0.006	8.813	$<10^{-4}$

*Table II.2.2 Relationships between ecological input data and Log(**T_{DG}**)/Log(**T_D**) (HP)*

n=770 datasets

Variable	R^2	*P*
m	0.047	$<10^{-4}$
N	0.010	$<10^{-4}$
Log(K_t)	NA	ns
F	0.071	$<10^{-4}$
P	0.233	$<10^{-4}$
C_p	0.047	$<10^{-4}$

3) The ratio (T_{DG}/T_G)

*Table II.3.1 Relationships between ecological input data and Log(**T_{DG}**)/Log(**T_G**) (GLM)*

n=794 datasets, overall adjusted R^2=0.19

Variable	Estimate	*sd*	*T*	*P*
m	-8.566	5.100	-1.679	ns
N	0.149	0.019	7.882	$<10^{-4}$
Log(K_t)	-1.744	0.169	-10.298	$<10^{-4}$
F	-6.353	0.964	-6.589	$<10^{-4}$
P	-1.283	3.848	-0.333	ns
C_p	-0.060	0.384	-0.156	ns

*Table II.3.2 Relationships between ecological input data and Log(**T_{DG}**)/Log(**T_G**) (HP)*

n=794 datasets

Variable	R^2	*P*
m	NA	ns
N	0.058	$<10^{-4}$
$\text{Log}(K_t)$	0.091	$<10^{-4}$
F	0.038	$<10^{-4}$
P	NA	ns
C_p	NA	ns

III. Appendix S3: Linear relationships between the residuals of regression models involving T_D, T_G and T_{DG} and ecological input data

In the following Generalized Linear Model (GLM) tables, estimates of the coefficients of regression are provided with standard errors (*sd*), Student statistics (*T*) and p-value (*P*). In hierarchical partitioning (HP) tables, the independent proportion of variance explained by each explanatory variable (R^2) is provided with a p-value (P) obtained from a randomization test (1000 iterations, see Mac Nally 2002, Walsh & Mac Nally 2003).

The dependent variables were the residuals of univariate regression models involving T_D, T_G and T_{DG}. Explanatory variables were the dispersal rate (*m*), the overall carrying capacity (K_t), number of patches (*N*), individual fecundity (*F*), per generation frequency of perturbations (*P*) and coefficient of correlation of perturbations among patches (C_p). Interactions between explanatory variables were not considered. In all cases, T_D, T_G and T_{DG} were log-transformed. Other data transformations are directly indicated in tables.

1) Residuals of the regression of Log(T_{DG}) by Log(T_D)

T_{DG} and T_D were positively correlated and T_D explained 68% of the variance in T_{DG}. The analysis of residuals is presented below.

Table III.1.1 Relationships between ecological input data and residuals (GLM)

n=770 datasets, overall adjusted R^2=0.26

Variable	Estimate	*sd*	*T*	*P*
m	-2.487	0.470	-5.287	$<10^{-4}$
N	-0.003	0.002	-1.617	ns
Log(K_t)	0.159	0.014	10.956	$<10^{-4}$

F	0.240	0.092	2.614	0.009
P	2.323	0.362	6.420	$<10^{-4}$
C_p	0.261	0.038	6.961	$<10^{-4}$

Table III.1.2 Relationships between ecological input data and residuals (HP)

n=770 datasets

Variable	R^2	*P*
m	0.012	ns
N	0.081	$<10^{-4}$
Log(K_t)	0.434	$<10^{-4}$
F	0.031	0.001
P	0.045	0.009
C_p	0.011	ns

2) Residuals of the regression of Log(T_{DG}) by Log(T_G)

T_{DG} and T_G were positively correlated and T_G explained 65% of the variance in T_{DG}. The analysis of residuals is presented below.

Table III.2.1 Relationships between ecological input data and residuals (GLM)

n=796 datasets, overall adjusted R^2=0.17

Variable	Estimate	*sd*	*T*	*P*
m	-1.854	0.472	-3.925	$<10^{-4}$
N	0.011	0.002	6.363	$<10^{-4}$
Log(K_t)	-0.060	0.016	-3.844	0.0001
F	1.468	0.089	16.434	$<10^{-4}$

P	-4.314	0.357	-12.101	$<10^{-4}$
C_p	-0.144	0.036	-4.037	$<10^{-4}$

Table III.2.2 Relationships between ecological input data and residuals (HP)

n=796 datasets

Variable	R^2	P
m		ns
N		ns
$\mathrm{Log}(K_t)$		ns
F	0.228	$<10^{-4}$
P	0.121	$<10^{-4}$
C_p		ns

IV. Complementary results (use of an alternative protocol to model environmental perturbations)

In the results presented below, environmental perturbations were assumed to reduce the local carrying capacities of patches for the demographic and demo-genetic models (the genetic model was as in the main results).

In the following Survival model tables, estimates of the coefficients of regression are provided with standard errors (*sd*), Z statistics (Z) and p-value (*P*). In generalized linear model (GLM) tables, estimates of the coefficients of regression are provided with standard errors (*sd*), Student statistics (*T*) and p-value (*P*). In hierarchical partitioning (HP) tables, the independent proportion of variance explained by each explanatory variable (R^2) is provided with a p-value (P) obtained from a randomization test (1000 iterations, see Mac Nally 2002, Walsh & Mac Nally 2003).

The dependent variables were the demographic (T_D), genetic (T_G) or demo-genetic (T_{DG}) median times to extinction. Explanatory variables were the dispersal rate (*m*), the overall carrying capacity (K_t), number of patches (*N*), individual fecundity (*F*), per generation frequency of perturbations (*P*) and coefficient of correlation of perturbations among patches (C_p). Interactions between explanatory variables were not considered. In all cases, T_D, T_G and T_{DG} were log-transformed. Other data transformations are directly indicated in tables.

A. Constancy of extinction rates over time

Exponential and Weibull survival models assuming right censored extinction events were fitted to examine the relationship between extinction times and ecological input parameters, considering all 1476 scenarios (Cox and Oakes 1984). The Weibull and exponential models

were compared to describe whether extinction rates were constant over time or not (Crawley, 2007). The analysis indicated that extinction rates were not constant across time for the demographic (T_D) and demo-genetic (T_{DG}) median times to extinction (Table *IV*.A.1.1 below). In all cases, extinction rates increased with time (the Log(scale) estimate was significantly different from zero and negative in all Weibull regressions, see Tables *IV.B.1.1* and *IV.B.2.1* below). All subsequent survival models (subsection B) are based on a Weibull distribution of extinction times.

Table IV.A.1.1 Comparison between exponential and Weibull models (ANOVA) for T_D and T_{DG}.

Dependant variable	χ^2	d.f.	P
T_D	572.7	1	$<10^{-4}$
T_{DG}	1022.6	1	$<10^{-4}$

B. *Linear relationships between times to extinction (T_D and T_{DG}) and ecological input data*

B.1) The demographic time to extinction (T_D)

Table IV.B.1.1 Relationships between ecological input data and Log($\mathbf{T_D}$) (Survival model)

n=1476 datasets; model log-likelihood=-2126; χ^2=1281.13; d.f.=6; P<10^{-4}

Variable	Estimate	*sd*	*Z*	*P*
m	5.38	0.47	11.50	$<10^{-4}$
N	-0.02	0.00	-10.50	$<10^{-4}$
Log(K_t)	0.38	0.015	25.84	$<10^{-4}$
F	1.11	0.08	13.45	$<10^{-4}$
P	-7.50	0.26	-28.62	$<10^{-4}$

C_p	-0.19	0.03	-5.66	$<10^{-4}$
Log(scale)	-0.87	0.03	-29.91	$<10^{-4}$

*Table IV.B.1.2 Relationships between ecological input data and Log(**T_D**) (GLM)*

n=703 datasets, overall adjusted R^2=0.64

Variable	Estimate	*sd*	*T*	*P*
m	12.140	1.427	8.508	$<10^{-4}$
N	-0.099	0.005	-19.547	$<10^{-4}$
Log(K_t)	1.397	0.046	30.228	$<10^{-4}$
F	3.132	0.267	11.713	$<10^{-4}$
P	-8.651	0.939	-9.212	$<10^{-4}$
C_p	-0.985	0.109	-9.061	$<10^{-4}$

*Table IV.B.1.3 Relationships between ecological input data and Log(**T_D**) (HP)*

n=703 datasets

Variable	R^2	*P*
m	0.008	0.010
N	0.173	$<10^{-4}$
Log(K_t)	0.383	$<10^{-4}$
F	0.046	$<10^{-4}$
P	0.024	$<10^{-4}$
C_p	0.015	$<10^{-4}$

B.2) The demo-genetic time to extinction (T_{DG})

*Table IV.B.2.1 Relationships between ecological input data and Log(**T_{DG}**) (Survival model)*

n=1476 datasets; model log-likelihood=-2540.4; χ^2=1255.85; d.f.=6; P<10^{-4}

Variable	Estimate	*sd*	*Z*	*P*
m	1.71	0.31	5.45	<10^{-4}
N	-0.01	0.001	-6.07	<10^{-4}
Log(K_t)	0.19	0.01	19.32	<10^{-4}
F	1.04	0.06	16.52	<10^{-4}
P	-5.48	0.20	-27.60	<10^{-4}
C_p	0.39	0.03	13.54	<10^{-4}
Log(scale)	-0.92	0.02	-40.69	<10^{-4}

*Table IV.B.2.2 Relationships between ecological input data and Log(**T_{DG}**) (GLM)*

n=1122 datasets, overall adjusted R^2=0.74

Variable	Estimate	*sd*	*T*	*P*
m	5.050	0.583	8.659	<10^{-4}
N	-0.041	0.002	-18.111	<10^{-4}
Log(K_t)	0.749	0.018	40.573	<10^{-4}
F	4.504	0.115	39.106	<10^{-4}
P	-3.104	0.396	-7.843	<10^{-4}
C_p	-0.328	0.048	-6.815	<10^{-4}

*Table IV.B.2.3 Relationships between ecological input data and Log(**T_{DG}**) (HP)*

n=1122 datasets

Variable	R^2	*P*
m	0.006	0.003

N	0.081	$<10^{-4}$
Log(K_t)	0.324	$<10^{-4}$
F	0.318	$<10^{-4}$
P	0.014	$<10^{-4}$
C_p	0.004	0.030

C: Linear relationships between the ratios of the times to extinction and ecological input data

C.1) The ratio (T_G/T_D)

Table IV.C.1.1 Relationships between ecological input data and Log($\mathbf{T_G}$)/Log($\mathbf{T_D}$) (GLM)

n=689 datasets, overall adjusted R^2=0.79

Variable	Estimate	*sd*	*T*	*P*
m	0.865	0.118	7.308	$<10^{-4}$
N	-0.011	0.000	-26.899	$<10^{-4}$
Log(K_t)	0.125	0.004	32.105	$<10^{-4}$
F	0.494	0.022	22.720	$<10^{-4}$
P	0.249	0.076	3.270	0.001
C_p	0.106	0.009	12.076	$<10^{-4}$

Table IV.C.1.2 Relationships between ecological input data and Log($\mathbf{T_G}$)/Log($\mathbf{T_D}$) (HP)

n=689 datasets

Variable	R^2	*P*
m	0.010	0.001
N	0.225	$<10^{-4}$
Log(K_t)	0.292	$<10^{-4}$

F	0.136	$<10^{-4}$
P	0.024	$<10^{-4}$
C_p	0.103	$<10^{-4}$

C.2) The ratio (T_{DG}/T_D)

Table IV.C.2.1 Relationships between ecological input data and Log(T_{DG})/Log(T_D) (GLM)

n=703 datasets, overall adjusted R^2=0.35

Variable	Estimate	*sd*	*T*	*P*
m	-0.693	0.101	-6.885	$<10^{-4}$
N	0.004	0.000	12.289	$<10^{-4}$
Log(K_t)	-0.036	0.003	-11.084	$<10^{-4}$
F	0.092	0.019	4.850	$<10^{-4}$
P	0.461	0.066	6.953	$<10^{-4}$
C_p	0.063	0.008	8.254	$<10^{-4}$

Table IV.C.2.2 Relationships between ecological input data and Log(T_{DG})/Log(T_D) (HP)

n=703 datasets

Variable	R^2	*P*
m	0.024	$<10^{-4}$
N	0.121	$<10^{-4}$
Log(K_t)	0.084	$<10^{-4}$
F	0.043	$<10^{-4}$
P	0.038	$<10^{-4}$
C_p	0.050	$<10^{-4}$

C.3) The ratio (T_{DG}/T_G)

*Table IV.C.3.1 Relationships between ecological input data and Log(**T_{DG}**)/Log(**T_G**) (GLM)*

n=989 datasets, overall adjusted R^2=0.19

Variable	Estimate	*sd*	*T*	*P*
m	-8.519	4.431	-1.923	0.05
N	0.146	0.016	8.885	$<10^{-4}$
Log(K_t)	-1.711	0.148	-11.565	$<10^{-4}$
F	-6.210	0.839	-7.398	$<10^{-4}$
P	1.045	2.881	0.363	ns
C_p	0.021	0.354	0.060	ns

*Table IV.C.3.2 Relationships between ecological input data and Log(**T_{DG}**)/Log(**T_G**) (HP)*

n=989 datasets

Variable	R^2	*P*
m	0.003	ns
N	0.059	$<10^{-4}$
Log(K_t)	0.092	$<10^{-4}$
F	0.038	$<10^{-4}$
P	0.000	ns
C_p	0.002	ns

References for Additional file 1

Cox, D. & Oakes, D. (1984). *Analysis of Survival Data*. Chapman & Hall, London, UK.

Crawley, M.J. (2007*). The R Book.* John Wiley & Sons Ltd., Chichester, West Sussex, UK.

Mac Nally, R. (2002). Multiple regression and inference in ecology and conservation biology: further comments on identifying important predictor variables. *Biodivers. Conserv.*, 11, 1397–1401.

Walsh, C. & Mac Nally, R. (2003). The hier.part package. Hierarchical Partitioning. R project for statistical computing. URL: http://cran.r-project.org/.
