# Supplementary material for: Find the weakest link. A comparison between demographic, genetic and demo-genetic metapopulation extinction times
Source: BMC Evol Biol. 2011 Sep 19;11:260. doi: 10.1186/1471-2148-11-260 (PMC3185286; doi:10.1186/1471-2148-11-260)

Additional file 2. ***Sensitivity of fragmentation results to genetic parameters (U, s and h)***

Genetic (left panel) and demo-genetic (right) median extinction times as functions of the level of metapopulation fragmentation (*N*). Extinction times are presented for different per generation zygotic mutation rates (*U*, with *s* fixed to 0.05 and *h* fixed to 0.35, A and B panels), different average coefficients of selection (*s*, with *U* fixed to 1.0 and *h* fixed to 0.35, C and D panels) and different average coefficients of dominance (*h*, with *U* fixed to 1.0 and *s* fixed to 0.05, E and F panels). In all cases, environmental perturbations occur and act independently among patches. m=0.01; K_t,=250; F=1.1; P=0.05; C_p=0.

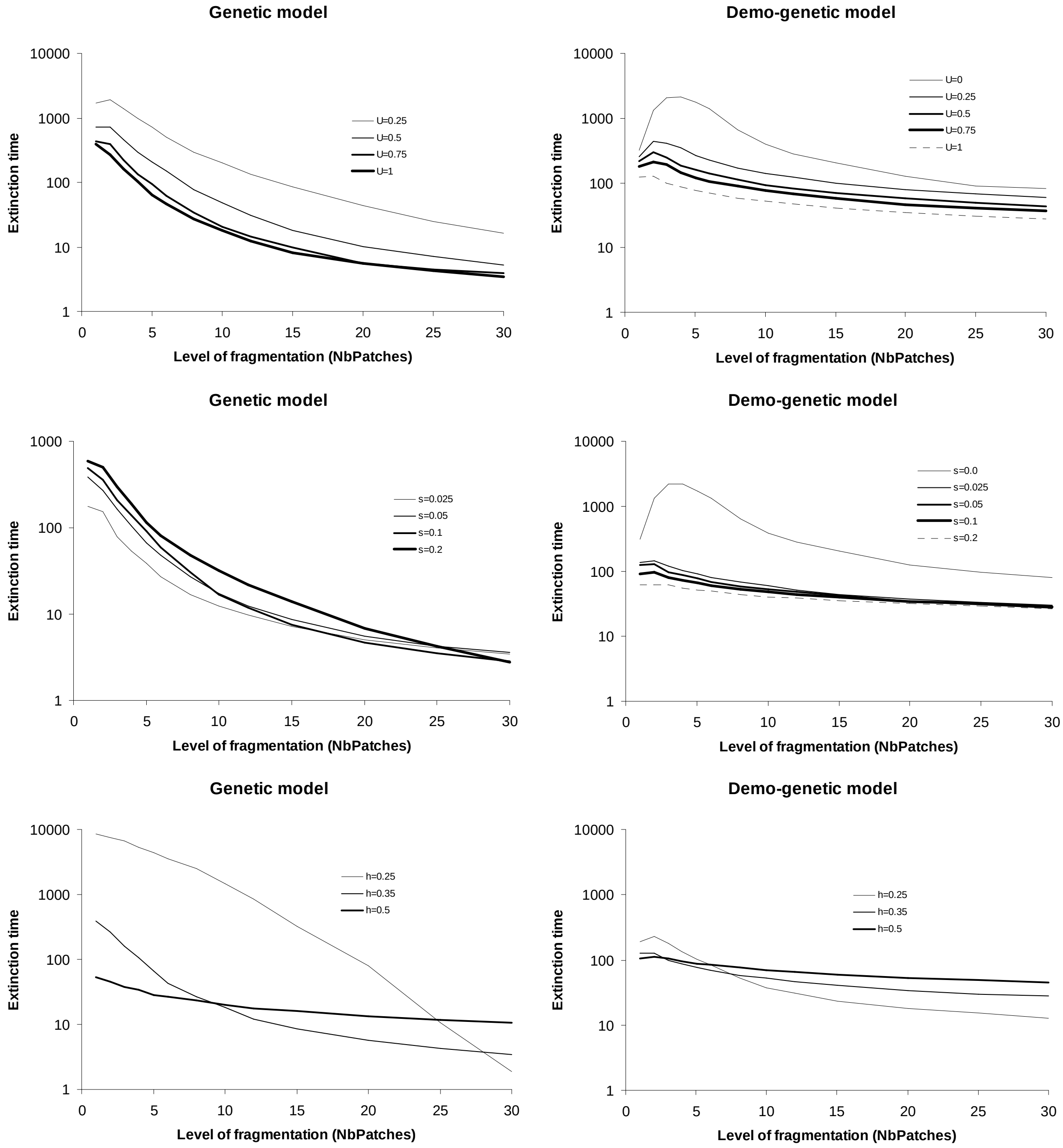

Supplement: Additional file 2 — Sensitivity of fragmentation results to genetic parameters (U, s and h). [file 1471-2148-11-260-S2.PDF]
