# Supplementary material for: Find the weakest link. A comparison between demographic, genetic and demo-genetic metapopulation extinction times
Source: BMC Evol Biol. 2011 Sep 19;11:260. doi: 10.1186/1471-2148-11-260 (PMC3185286; doi:10.1186/1471-2148-11-260)

Additional file 3. ***Effect of fragmentation on extinction times: regime of spatially correlated perturbations***

Demographic, genetic and demo-genetic median extinction times as functions of the level of metapopulation fragmentation (*N*). Extinction times are presented for different dispersal rates (*m*, ranging from 0 to 0.1, K_t fixed to 250, left panel) and different overall metapopulation carrying capacities (K_t, ranging from 50 to 1000, *m* fixed to 0.01, right panel). Continuous lines: low frequency of environmental perturbations (*P*=0.05); dotted lines: high frequency of environmental perturbations (*P*=0.15). In all cases, environmental perturbations occur and are fully correlated among patches. *F*=1.1.

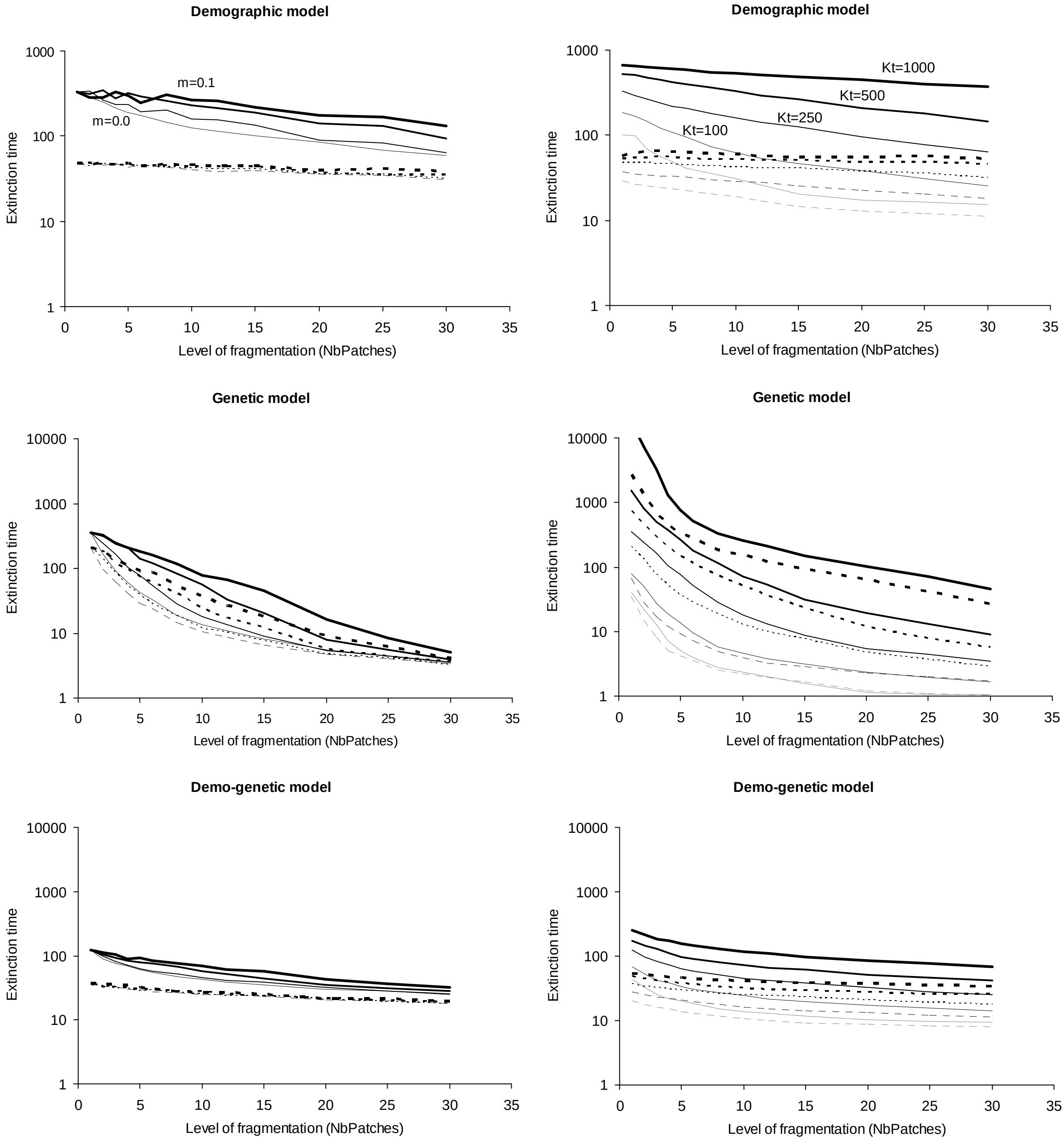

Supplement: Additional file 3 — Effect of fragmentation on extinction times: regime of spatially correlated perturbation. [file 1471-2148-11-260-S3.PDF]
