# Supplementary material for: Find the weakest link. A comparison between demographic, genetic and demo-genetic metapopulation extinction times
Source: BMC Evol Biol. 2011 Sep 19;11:260. doi: 10.1186/1471-2148-11-260 (PMC3185286; doi:10.1186/1471-2148-11-260)

Additional file 4. ***Effect of fragmentation on extinction times: complementary results (use of an alternative protocol to model environmental perturbations)***

Demographic, genetic and demo-genetic median extinction times as functions of the level of metapopulation fragmentation (*N*). In this figure, environmental perturbations were assumed to reduce the local carrying capacities of patches for the demographic and demo-genetic models (the genetic model was as in the main results). Extinction times are presented for different dispersal rates (*m*, ranging from 0 to 0.1, K_t fixed to 250). Continuous lines: low frequency of environmental perturbations (*P*=0.05); dotted lines: high frequency of environmental perturbations (*P*=0.15). In all cases, perturbations occur and act independently among patches. *F*=1.1.

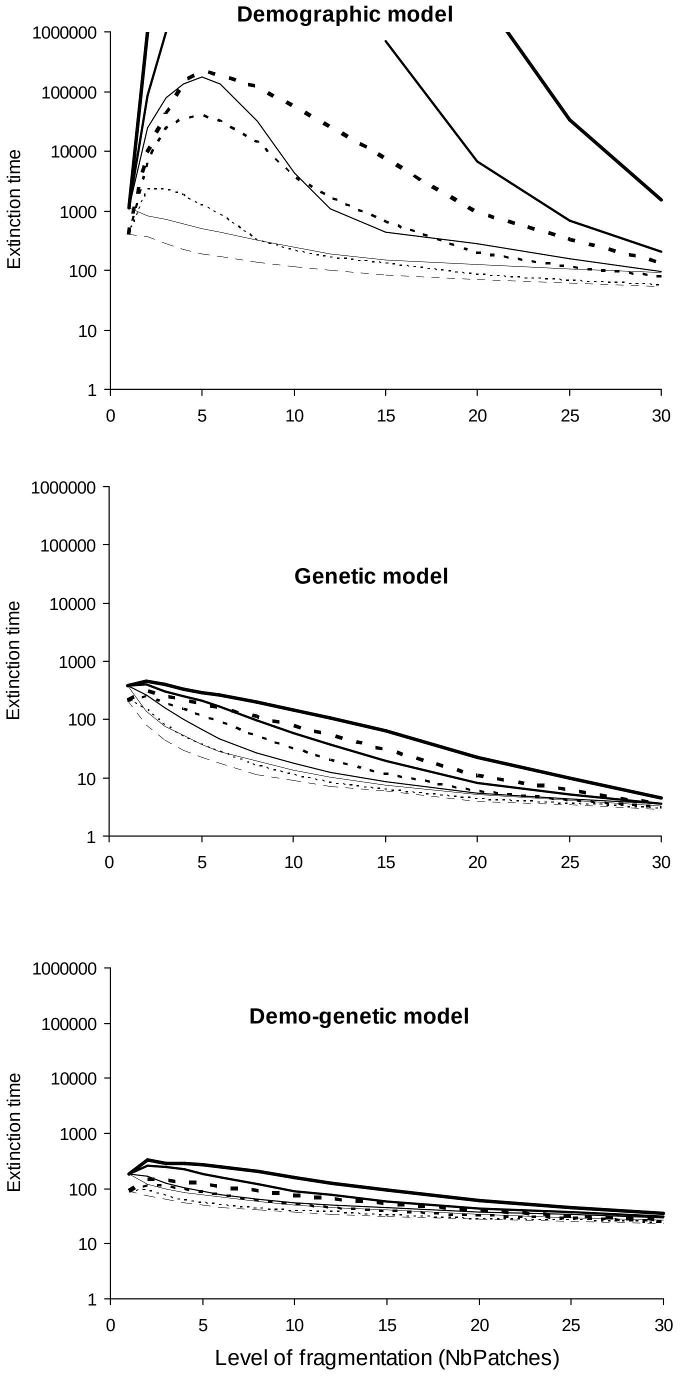

Supplement: Additional file 4 — Effect of fragmentation on extinction times: complementary results (use of an alternative protocol to model environmental perturbations). [file 1471-2148-11-260-S4.PDF]
