# Supplementary material for: Find the weakest link. A comparison between demographic, genetic and demo-genetic metapopulation extinction times
Source: BMC Evol Biol. 2011 Sep 19;11:260. doi: 10.1186/1471-2148-11-260 (PMC3185286; doi:10.1186/1471-2148-11-260)

Additional file 5. *Fitness and population size reductions: an illustration*

Changes in mean replacement rate and population size with the genetic (left panel) and demo-genetic (right) models: single population trajectories. Median metrics are computed over 250 trajectories, but only 30 trajectories are presented for clarity. m=0.01; K_t=50; N=10; F=1.5; P=0; C_p=NA.

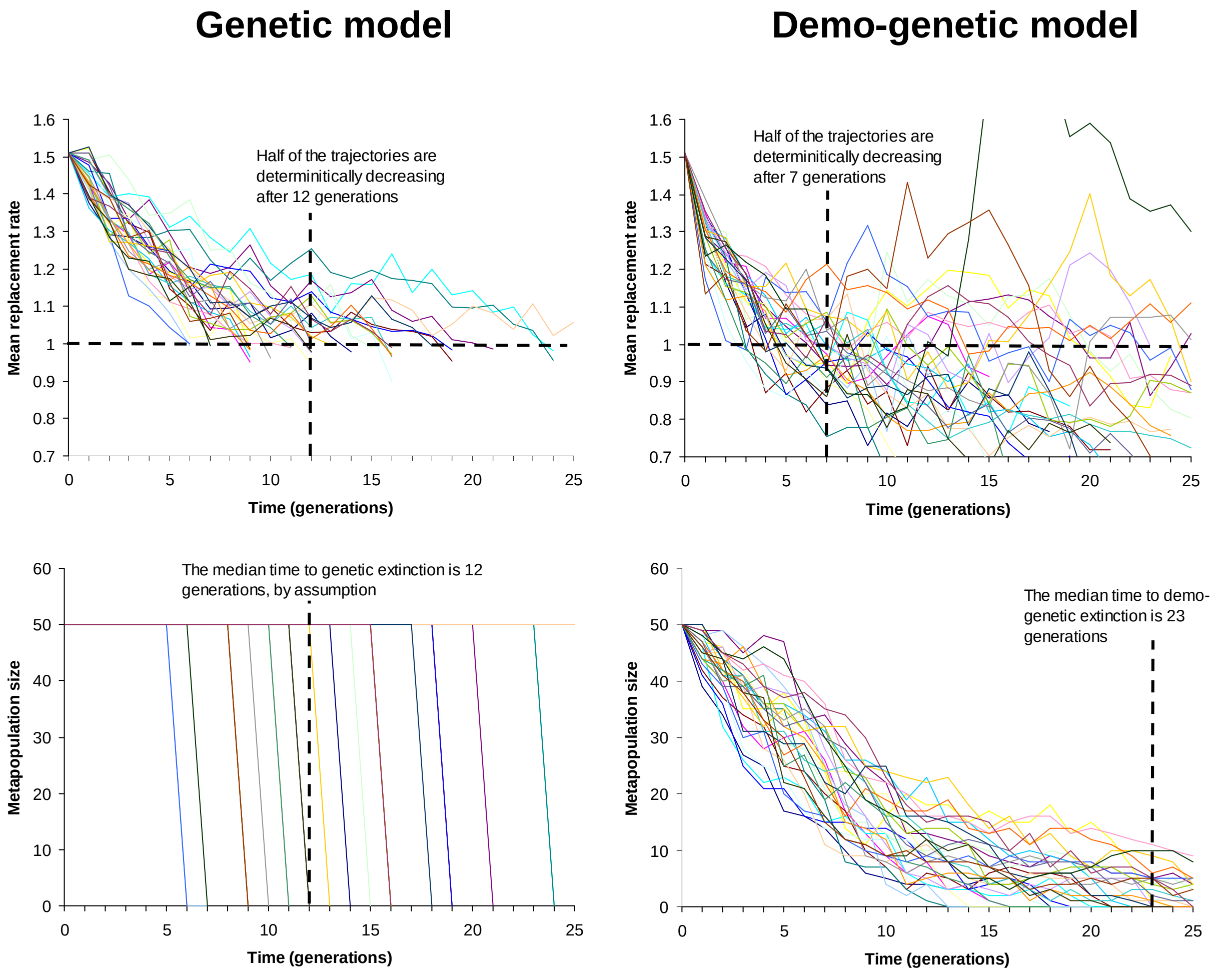

Supplement: Additional file 5 — Fitness and population size reductions: an illustration. [file 1471-2148-11-260-S5.PDF]
